# RETRACTION: The Z‐Plasty Contributes to The Coalescence of a Chronic Non‐Healing Wound

**DOI:** 10.1111/iwj.70648

**Published:** 2025-04-23

## Abstract

**RETRACTION:** ZhangX. , WangG. , SunY. , DinP. , YangX. , and ZhaoZ. , “The Z‐Plasty Contributes to The Coalescence of a Chronic Non‐Healing Wound,” International Wound Journal 18, no. 6 (2021): 796–804, 10.1111/iwj.13583.33733609 PMC8613384

The above article, published online on 17 March 2021, in Wiley Online Library (http://onlinelibrary.wiley.com/), has been retracted by agreement between the journal Editor in Chief, Professor Keith Harding; and John Wiley & Sons Ltd. Following an investigation by the publisher, all parties have concluded that this article was accepted solely on the basis of a compromised peer review process. The editors have therefore decided to retract the article. The authors did not respond to our notice regarding the retraction.

**RETRACTION:** X. Zhang , G. Wang , Y. Sun , P. Din , X. Yang , and Z. Zhao , “The Z‐Plasty Contributes to The Coalescence of a Chronic Non‐Healing Wound,” International Wound Journal 18, no. 6 (2021): 796–804, 10.1111/iwj.13583.33733609 PMC8613384

The above article, published online on 17 March 2021, in Wiley Online Library (http://onlinelibrary.wiley.com/), has been retracted by agreement between the journal Editor in Chief, Professor Keith Harding; and John Wiley & Sons Ltd. Following an investigation by the publisher, all parties have concluded that this article was accepted solely on the basis of a compromised peer review process. The editors have therefore decided to retract the article. The authors did not respond to our notice regarding the retraction.

